# Aberrant AFP expression characterizes a subset of hepatocellular carcinoma with distinct gene expression patterns and inferior prognosis

**DOI:** 10.7150/jca.31435

**Published:** 2020-01-01

**Authors:** Wei Chen, Jianjun Peng, Jinning Ye, Weigang Dai, Guanghua Li, Yulong He

**Affiliations:** Department of Gastrointestinal Surgery, First Affiliated Hospital of Sun Yat-sen University, 510080 Guangzhou, Guangdong province, People's Republic of China.

**Keywords:** AFP expression, liver cancer, De-methylation

## Abstract

**Background** Serum tumor markers are ubiquitously used in the clinic for cancer screening. However, the mechanisms accounting for the elevated levels of the serum tumor markers remain to be explored.

**Methods** We performed a pan-cancer analysis of serum alpha-fetoprotein (AFP), carcinoembryonic antigen (CEA) and prostate-specific antigen (PSA). The relation between concentration of serum tumor markers and the expression of their coding genes was assessed. Then the expression of AFP and its genomic background in hepatocellular carcinoma (liver cancer) was studied.

**Results** High expression of AFP mRNA was found mainly in liver cancer. In gastric cancer, breast cancer and lung cancer, high AFP mRNA expression was also discovered occasionally. In liver cancer patients, serum AFP levels correlated significantly with AFP mRNA expression in cancer tissues (r = 0.72, p = 1.6e-45). Whole transcriptome analysis revealed that serum AFP levels clearly separated liver cancer into two classes with distinct expression profiles according to PCA analysis. Gene co-expression analysis revealed that AFP expression was connected to a module enriched with genes accounting for cell cycle and cell proliferation regulation. In addition, high AFP expression was associated with the molecular classification of liver cancer, including iCluster (Chi-square: 16.86, P = 0.0002). Methylation analysis revealed de-methylation of AFP promoter occurred in some liver cancer tissues, which was significantly related to AFP mRNA expression. Survival analysis indicated high serum AFP levels was prognostic of poorer survival of the liver cancer patients (Log-rank test: p = 0.046). This was confirmed by an independent dataset in which liver cancer patients with high serum AFP also had poorer survival (Log-rank test: p = 0.024).

**Conclusion** High expression of AFP defined a subtype of liver cancer with distinct gene expression profiles and clinical features. De-methylation of cytosine from CpG di-nucleotides in AFP promoter may be the cause of AFP re-expression in adult human liver cancer tissue.

## Introduction

Serum tumor markers are essential noninvasive tools for the screening and diagnosis of various cancer types. Many serum tumor markers have been studied and approved for use in the clinic. For example, alpha-fetoprotein (AFP) is widely used for the diagnosis of hepatocellular carcinoma (liver cancer). In addition, positive AFP could also be encountered in other cancer types^1^-^4^. The carcinoembryonic antigen (CEA) is used to assist in the diagnosis of cancers that arise from gastrointestinal tract^5^. The prostate-specific antigen (PSA), which is a prostate differentiation marker, is exclusively used for screening of prostate cancer. Other markers, including CA15-3, CA125, CA19.9, et.al. are also widely used for the screening of various cancer types^6^.

AFP is the main component of mammalian fetal serum. It is synthesized by visceral endoderm of the yolk sac and by fetal liver. After birth AFP level decreases dramatically in blood. However, AFP synthesis can be reactivated in liver tumors and germinogeneous teratoblastomas. As a result, serum AFP is an important marker for liver tumors and is widely used in clinical practice 7. Although aberrant AFP expression is mainly found in liver cancer, it could also be found occasionally in gastric cancer^4^,^8^, breast cancer 2, lung cancer^1^,^3^, pancreatic cancer 9. This raises an interesting question as why AFP is activated in such tumors. The mechanisms leading to aberrant expression of AFP in liver cancer and other types of tumors are not clear yet.

Carcinoembryonic antigen (CEA) is a glycoprotein of about 200,000 Daltons in molecular weight. It is expressed in significant amounts during embryonic life, especially by the large intestine, and postnatally by carcinomas arising from this site. Many tumors of epithelial origin at other sites may also express CEA and are associated with elevated CEA levels in blood circulation 5. CEA level is often monitored in the management of colorectal cancer and its measurement before surgery is recommended by American Society of Clinical Oncology(ASCO). CEA level can be measured prior to surgery to predict prognosis. Besides, CEA can also be employed to assess response to treatment or after completion of therapy to monitor for recurrence^6^.

PSA is most frequently detected in prostate cancer. Positive correlation has been confirmed between the PSA level and tumor stage and volume^10^. Besides, PSA is an indicator for prostate cancer survival and recurrence^11^,^12^. Additionally, a serial test of PSA is valuable for monitoring the treatment efficacy 13.

Although serum tumor markers like AFP, CEA and PSA have been extensively studied and used in the clinic, questions regarding the mechanisms leading to their expression in cancer have not been well answered. This is exemplified by AFP, for which the molecular mechanisms leading to re-expression of AFP in adult human liver cancer and other tumor types is still unclear^7^. Knowing the source of these serum tumor markers may be helpful for us to understand the molecular mechanisms accounting for their aberrant expression, and to uncover the advantages as well as the limitations related to each of them. In addition, it may also be helpful in finding new serum markers for cancer diagnosis.

In this study, we tried to address the questions mentioned above using genomic data from large cohort of cancer patients. We compared the expression of the coding genes of AFP, CEA and PSA, and found that only AFP expression correlated with its serum levels in liver cancer patients. Further analysis revealed aberrant AFP re-expression actually represented a shift of cell transcriptome that involved many genes, especially those related to cell proliferation regulation. We then identified de-methylation of a CpG locus located at the promoter of AFP that may be accountable for the AFP re-activation.

## Method

### Patients and samples

Totally 4,666 tumor samples were included in the pan-cancer gene expression analysis of tumor markers. There were 421 liver cancer samples, 326 colon cancer samples and 549 prostate cancer samples. The details of patient and sample information could be found in the publications associated with each study^14^-^16^. The abbreviations for different datasets used are as follows: BRCA (Breast invasive carcinoma), STAD (Stomach adenocarcinoma), PAAD (Pancreatic adenocarcinoma), COAD (Colon adenocarcinoma), READ (Rectum adenocarcinoma), LIHC (Liver hepatocellular carcinoma), LUAD (Lung adenocarcinoma), LUSC (Lung squamous cell carcinoma), OV (Ovarian serous cystadenocarcinoma), and PRAD (Prostate adenocarcinoma).

For hepatocellular carcinoma dataset from TCGA (The Cancer Genome Atlas) study, all patients received surgical resection of their tumors. No other treatment, for example ablation, chemotherapy, or radiotherapy had been given to the patients before surgery. Surgical specimens of primary hepatocellular carcinoma and matched blood samples were collected for each patient. Totally 371 patients with full genomic data were included in this study. For clinical data analysis, patient information from the TCGA hepatocellular carcinoma publication was used, which contained 196 samples^16^. In another dataset GSE14520, hepatocellular carcinoma patients who underwent radical resection were enrolled. Tumor samples and adjacent normal liver tissues were subjected to microarray gene expression analysis. Only samples tested by Affymetrix HT Human Genome U133A Array were included in our analysis. Totally, there were 220 normal liver and 225 liver cancer tissues in this dataset.

### Data availability

Genomics data from TCGA project were downloaded using RTCGA package in R. The most recent version of RTCGA dataset was used (2016-01-28). For mRNA sequencing analysis, the RNASeq v2 data was used. For methylation analysis of TCGA data, the beta values of each CpG locus derived from Human Methylation 450 array platform was used. For survival analysis, a recently updated follow-up data processed by Liu J et.al. was used^17^. An independent data set profiling the whole methylome and transcriptome of three fetal liver and three adult liver tissues were downloaded from Gene Expression Omnibus (GEO) with accession IDs GSE69852 and GSE69713. Another dataset profiling a large cohort of liver cancer tissues and non-cancer tissues was also downloaded from GEO (GSE14520).

### Gene co-expression network analyses

Gene co-expression network analyses was performed using Co-Expression Modules identification Tool (CEMiTool), an R package that can identify and analyze co-expression modules in a fully automated manner. CEMiTool is featured by unsupervised gene filtering, automated parameter selection for identifying modules, enrichment and module functional analyses, as well as integration with interactome data to generate molecular interactions^18^.

### Gene methylation analysis

The whole genome methylation analysis was performed using Human Methylation 450 array platform. There are 4 probes targeting AFP gene, including one probe mapped onto the promoter region (cg10778295), two probes mapped onto around -5,000 from the transcription start site (cg02199826, cg03874137), and another probe mapped onto the interior of the gene (cg02387745). However, the probe inside AFP has no readings. Pearson correlation analysis was performed to assess relationship between methylation status of each probe with AFP expression.

### Statistical analysis

Statistical analysis was performed using R software. Human genome assembly hg19 was used as the reference genome. Liver cancer patients were separated into three groups based on serum AFP levels: high (>= 300 ng/ml), middle (< 300 ng/ml, >= 6 ng/ml), low (< 6 ng/ml). Survival plot was estimated using Kaplan-Meier method and groups were compared with log-rank test. For correlation analysis between the levels of markers in serum and tumor, serum marker levels were added by 1 and then log2 transformed. Pearson correlation analysis was then performed. For comparison of means of gene expression or methylation between two groups, t test was used.

## Results

### Expression of AFP, CEA and KLK3 across ten tumor types

**Figure 1:**
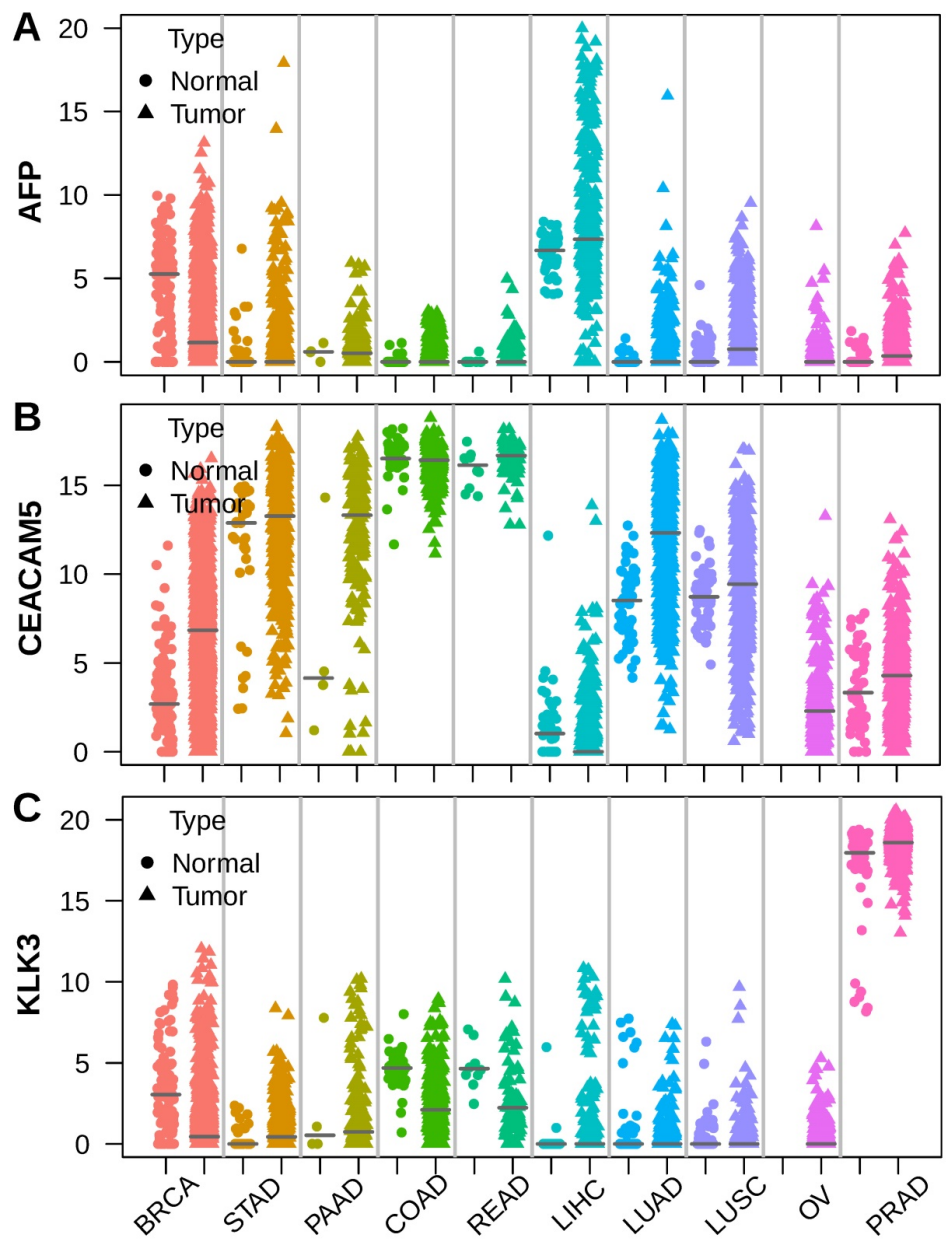
Stripchart showing the expression of AFP, CEA (CEACAM5) and PSA (KLK3) in tumor and adjacent normal tissues across ten selected tumor types. Dot represents normal tissue and triangle represents tumor tissue. Horizontal bar indicates median of gene expression. (A) AFP was expressed in both normal liver and liver cancer, with some liver cancer showing much higher AFP expression. (B) CEACAM5 was expressed at very high levels in both normal and tumor colorectal tissue. (C) KLK3 was expressed at very high levels in both normal prostate tissue and prostate cancer.

To unveil the roots of serum tumor markers, we selected AFP, CEA (encoded by CEACAM5)^19^ and PSA (encoded by KLK3)^20^, which are widely used in hepatocellular carcinoma (LIHC), colorectal cancer (COAD, READ) and prostate cancer (PRAD), respectively. We assessed the expression of these markers using RNA sequencing data across 10 major tumor types from TCGA^21^. AFP was highly expressed in a subset of liver cancer compared to normal liver. Apart from liver cancer, high expression of AFP was also observed in breast cancer (BRCA), gastric cancer (STAD) and lung cancer (LUAD, LUSC) sometimes (Figure 1A). The CEA coding gene CEACAM5 was highly expressed by normal colorectal epithelial and intermediately expressed by normal gastric and lung tissue. Colorectal cancer expressed similar levels of CEACAM5 as normal colorectal tissue. However, some gastric cancer, pancreatic cancer (PAAD), lung cancer and breast cancer also showed elevated CEACAM5 expression (Figure 1B). The PSA coding gene KLK3 was expressed at similar high levels in normal and tumor prostate tissue, but was never seen in other normal or tumor tissues (Figure 1C). These data suggested higher expression of AFP, CEACAM5 and KLK3 in tumors was a prerequisite for their presence in the serum of patients with specific cancer types.

### Serum AFP levels correlated with AFP gene expression in liver tumor

**Figure 2:**
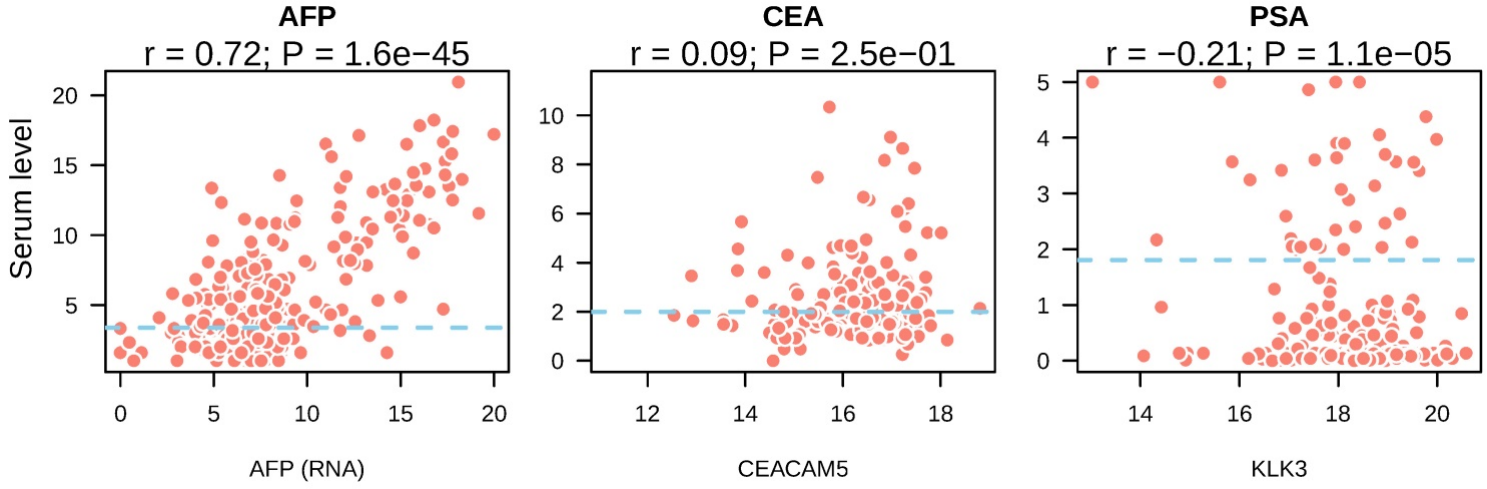
Correlation between serum tumor marker levels and the expression of their coding genes in tumor tissues. The mRNA expression of tumor marker coding genes in each tumor type was depicted in the x axis and serum tumor marker levels were shown in y axis. Blue dotted lines indicated the reference cutoff values of each tumor marker used in the clinic for cancer diagnosis. Both serum marker levels and mRNA expression levels were log2 transformed.

Next, we asked whether the presence of tumor markers in the bloodstream was resulted from high expression of these markers by tumors. We compared the expression of CEA, AFP and PSA in tumor tissue with their serum levels. In liver cancer, a strong correlation between AFP gene expression and serum levels was observed (r = 0.72, p = 1.6e-45) (Figure 2A). In colorectal cancer, CEACAM5 expression showed no correlation with its serum levels (r = 0.09, p = 0.25) (Figure 2B). In prostate cancer, KLK3 expression showed significant correlation with PSA levels from the serum of prostate cancer patients (r = -0.21, P = 1.1e-0.5) (Figure 2C). However, the coefficient of Pearson correlation was negative and p value was small, suggesting it may be a result of stochastic cause. These data suggested that high serum AFP in liver cancer patients was caused by re-activation and expression of AFP in liver tumors.

To validate these findings, we chose an independent dataset containing a large cohort of samples. Similarly, the expression of AFP were significantly higher in liver cancer than non-tumor liver tissue (t-test: p = 3.78e-24) (Supplement Figure 1). In addition, patients with higher serum AFP levels also had significantly higher AFP expression in the primary liver tumors (t-test: p = 7.92e-23) (Supplement Figure 1). These data confirmed that aberrant high AFP expression was observed in some liver cancer, and it was closely related to the high serum AFP levels in these patients.

### PCA analysis of liver cancer with gene expression data

**Figure 3:**
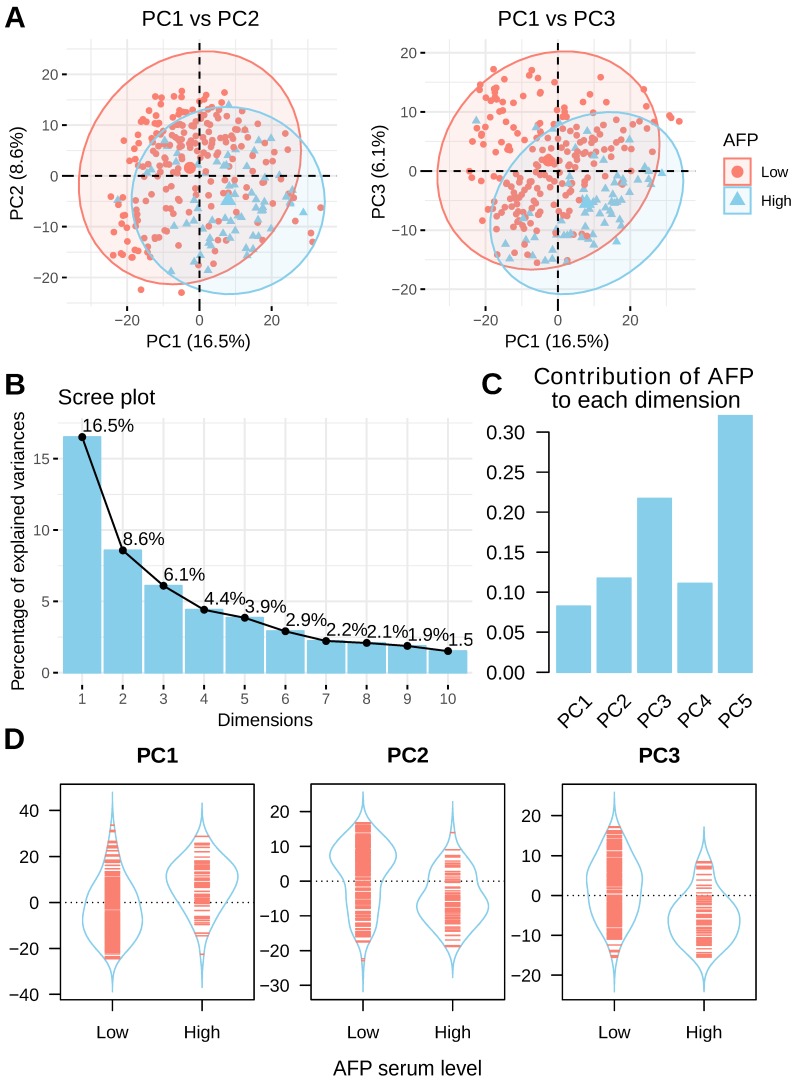
PCA analysis of liver cancer using whole transcriptome profiling data. (A) PCA analysis of liver cancer using top 1000 most variable genes across all liver cancer tissues. Patients with high serum AFP were labeled in blue and patients with low serum AFP were labeled in red. (B) The contribution of top 10 principals to total variance of all samples. (C) The contribution of AFP expression to top five components. (D) The coordinates of top three components for each sample as grouped by serum AFP levels of the patients.

Principal component analysis (PCA) was performed to assess the inner structure of liver cancer expression data. The distribution of liver cancer samples was plotted using the top three principal components and patients are colored by their serum AFP levels. A clear separation between high and low serum AFP patients could be seen (Figure 3A). This data suggested that AFP high expression may represent a subtype of liver cancer as defined by distinct transcriptome profiles. In total, the top three principal components in PCA accounted for 31.2% of total variance (16.5%, 8.6% and 6.1% for each) (Figure 3B). AFP contributed to 8.3%, 11.8% and 21.7% of top three components, respectively (Figure 3C). In addition, AFP was positively correlated with PC1, but negatively correlated with PC2 and PC3 (Figure 3D). In another dataset GSE14520, liver cancer patients with high and low serum AFP were also clearly separable as indicated by PCA analysis (Supplement Figure 2). These data revealed that AFP high expression liver cancer had distinct gene expression profiles compared with AFP low expression liver cancer.

### Gene co-expression analysis in liver cancer

**Figure 4:**
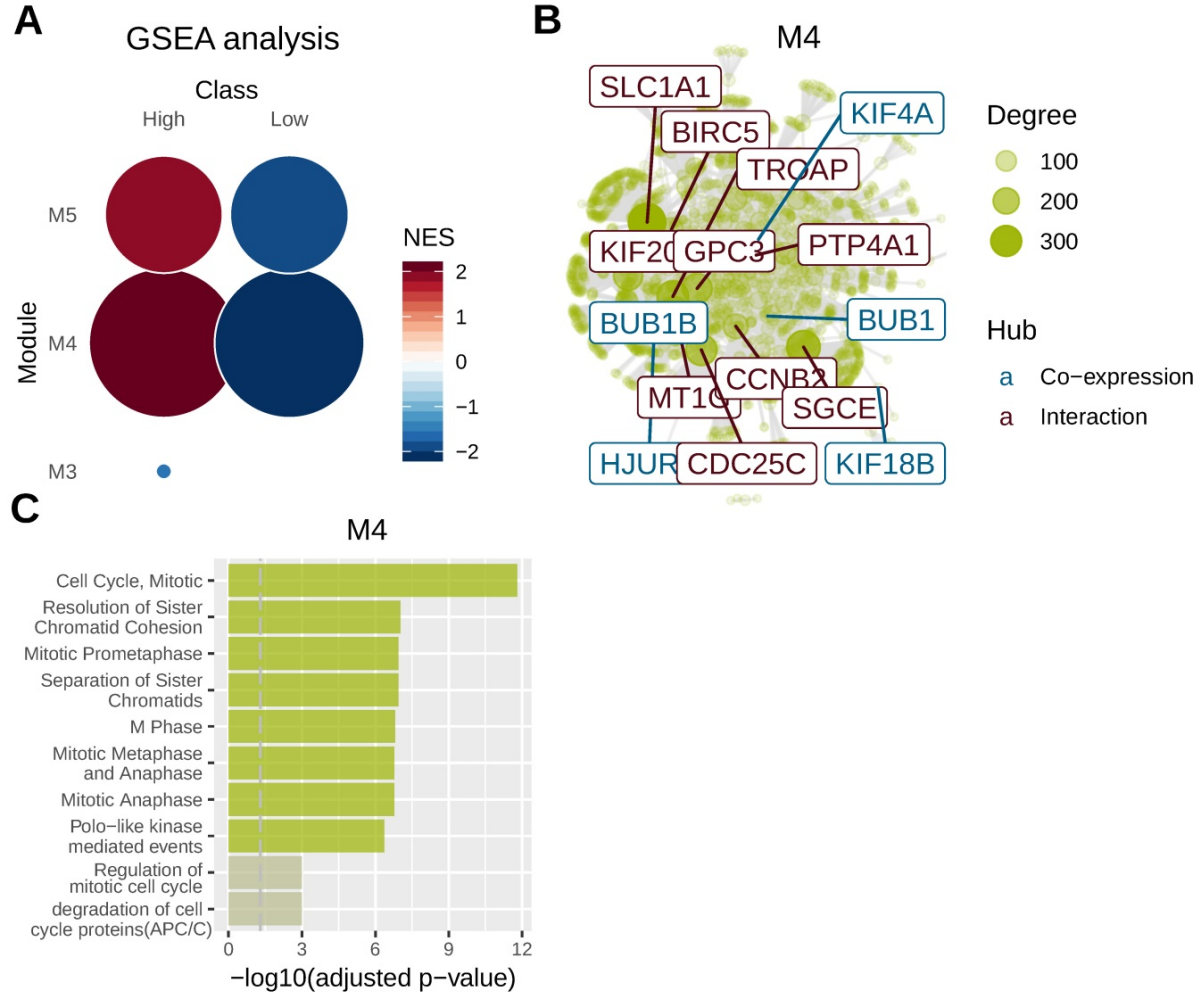
Gene co-expression analysis in liver cancer. Whole transcriptome data from TCGA liver cancer study was used for gene co-expression analysis. Tumor samples were grouped according to serum AFP levels. (A) Geneset enrichment analysis (GSEA) revealed M4 and M5 modules were enriched in AFP high expressing samples. (B) protein-protein interaction network analysis of genes in M4 module. Ten hub genes based on protein-protein interaction network (brown) and five hub genes based on co-expression analysis (blue) were revealed. The size of the node is proportional to its degree. (C) Geneset over-representation analysis of genes in M4 module.

To identify key genomic modules co-regulated with AFP, we performed gene co-expression analysis using mRNA sequencing data of liver cancer. Five gene modules were revealed for liver cancer and AFP belonged to M4 module. M4 and M5 module were significantly related to AFP high expressing liver cancer (Figure 4A). We then integrated co-expression information with protein-protein interaction data to identify main regulators and hubs in the module M4. Ten hub genes based on protein-protein interaction network were revealed and five more were discovered by gene co-expression analysis (Figure 4B). Notably, many of those hub genes were related to cell proliferation. For example, CDC25C directs dephosphorylation of cyclin B-bound CDC2 and triggers entry into mitosis^22^ . CCNB2 (Cyclin B2) is a member of the B-type cyclins, which are essential components of the cell cycle regulatory machinery^23^. PTP4A1 is a cell signaling molecule that plays regulatory roles in a variety of cellular processes, including cell proliferation and migration^24^. BIRC5 is a member of the inhibitor of apoptosis (IAP) gene family, which encode negative regulatory proteins that prevent apoptotic cell death 25. Consistently, gene-set over-representation analysis revealed that cell proliferation related gene-sets were significantly enriched in M4 module (Figure 4C). These data suggested high expression of AFP may be related to aberrant cell proliferation control of liver cancer.

### AFP re-expression was associated with de-methylation of AFP promoter

**Figure 5:**
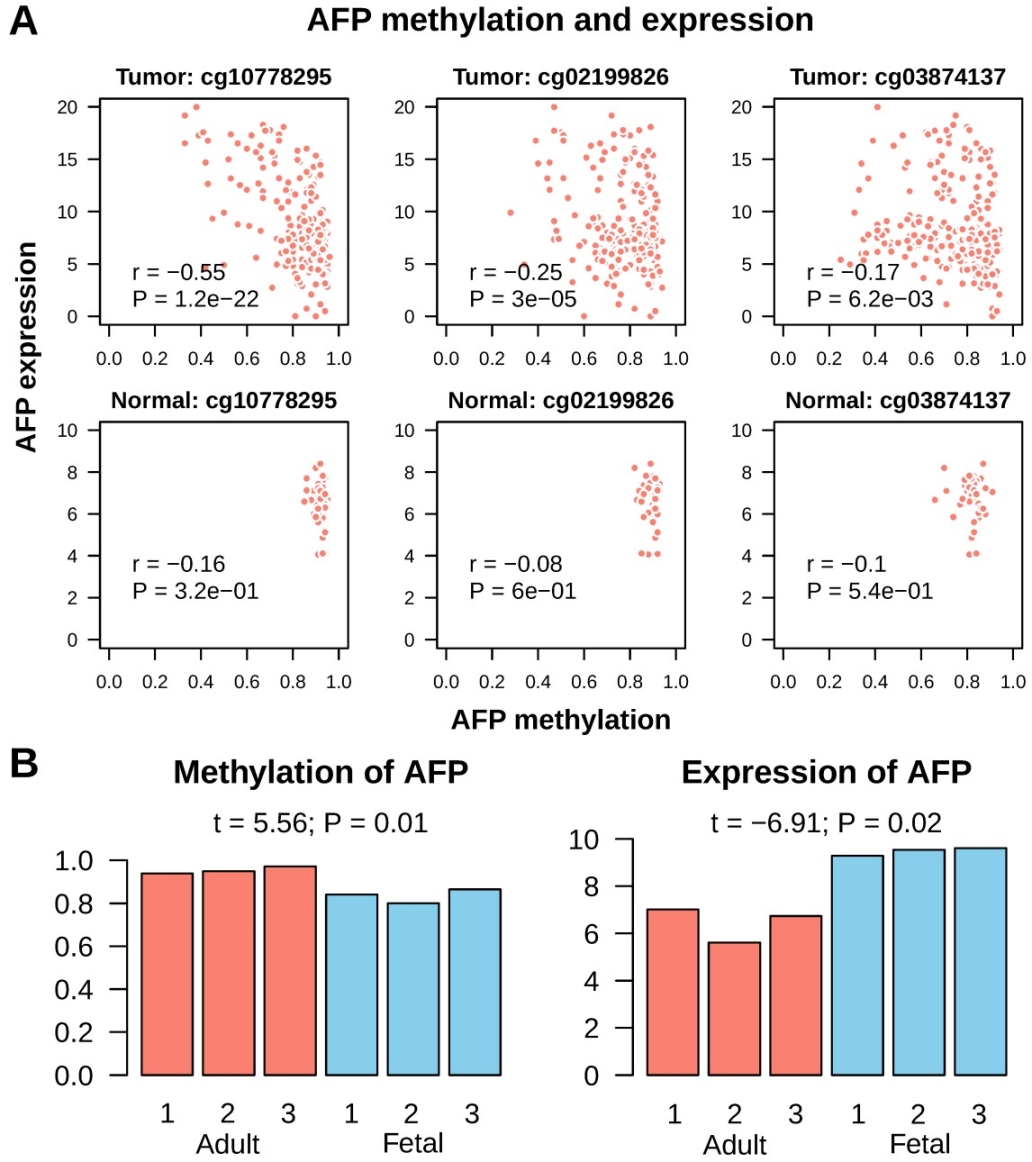
Methylation of AFP in normal liver and liver cancer. A, Correlation between AFP methylation and expression in normal adult liver and liver cancer tissue from TCGA study. Three CpGs located upstream of AFP transcription starting site were studied. Statistics in each figure are derived from Pearson correlation analysis. B, AFP methylation and expression in normal adult versus fetal liver. Data was downloaded from GEO datasets GSE69852 and GSE69713. Group means are compared using t test.

Next, we tried to find out what leaded to AFP re-expression in cancer. As hypo-methylation of promoter is a common mechanism for gene activation in cancer, we checked methylation of AFP in normal liver and liver cancer. In Infinium Human Methylation 450K Beadchip platform, which is widely used for whole genome methylation study, there are three probes mapped onto CpGs upstream of AFP transcription start site. All these three CpGs were invariably highly methylated in normal liver tissue (average beta values were cg10778295: 0.92±0.02; cg02199826: 0.89±0.03; cg03874137: 0.82±0.05) (Figure 5A). In addition, methylation of these CpGs had no correlation with AFP mRNA expression (Pearson correlation: all P > 0.1). In liver cancer, some tissues showed de-methylation in these three CpGs (average beta values were cg10778295: 0.83±0.13; cg02199826: 0.80±0.12; cg03874137: 0.75±0.15) (Figure 5A). All three CpGs showed significant de-methylation in liver cancer (t test: P < 1e-9). More importantly, methylation of cg10778295, a CpG located in the promoter of AFP (-824 from transcription start site), correlated very well with AFP mRNA expression (Pearson correlation: r = -0.55, P = 1.2e-22) (Figure 5A). We then validated this finding using fetal liver tissue, which normally expression AFP. In an independent data set, the whole methylome and transcriptome analysis were conducted in three fetal liver and three adult liver tissues (GSE69852 and GSE69713). Similar to the findings in liver cancer, fetal liver showed high methylation in cg10778295 and lower expression of AFP (t = 5.56, P = 0.01; t = -6.91, P = 0.02) (Figure 5B). These data demonstrated that de-methylation of AFP occurred in some liver cancer tissue, which may be accountable for re-activation of AFP transcription.

### Clinicopathologic and genomic relevance of AFP activation in cancer

**Figure 6:**
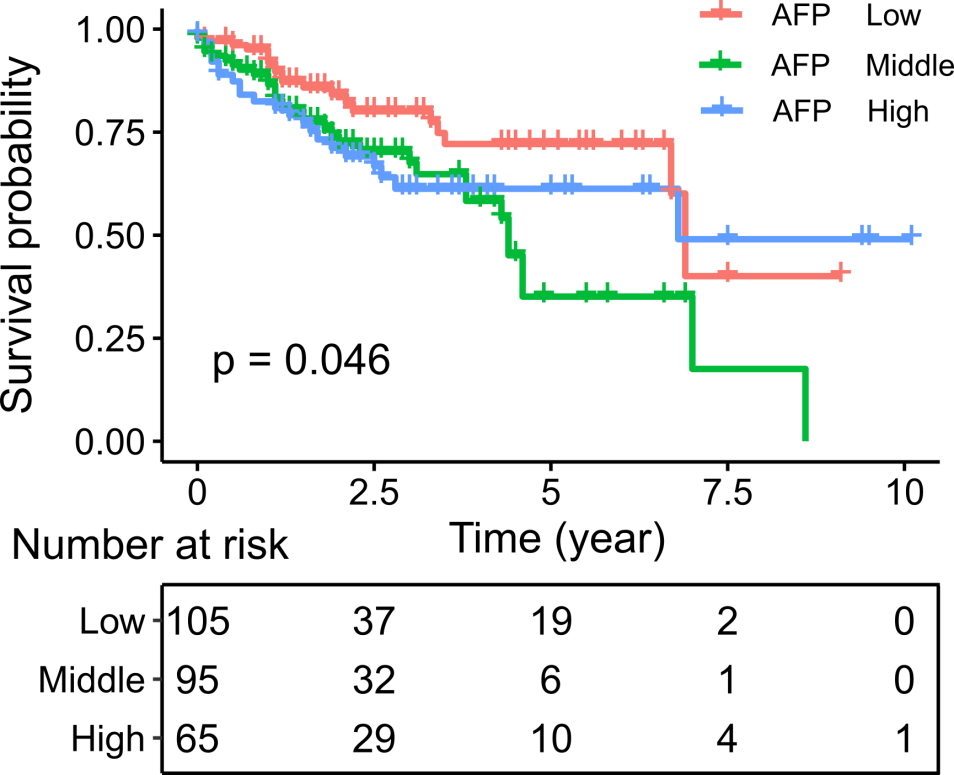
Kaplan-Meier plot showing the survival of liver cancer patients grouped by serum AFP levels. Survival rates were compared by log-rank test.

We then asked whether AFP expression had clinical significance. Survival analysis indicated poorer survival in liver cancer patient with middle and high serum AFP levels (Log-rank test: P = 0.046) (Figure 6). Similarly, in an independent dataset GSE14520, liver cancer patients with high serum AFP levels also had significantly worse overall survival than patients with low serum AFP levels (Log-rank test: p = 0.024) (Supplement Figure 3). To further assess the implications of high expression of AFP in liver cancer, we performed a thorough correlation study between serum AFP and clinicopathological features of the patients from TCGA study. Higher serum AFP level was associated with higher tumor grade (Chi-square: 8.07, P = 0.018) (Table 1). It also tended to correlate with more advanced tumor stage (Chi-square: 5.76, P = 0.056) (Table 1). Serum AFP level was not associated with gene mutations frequently found in liver cancer, including TP53 mutation, CTNNB1 mutation and TERT promoter mutation (Table 1).

Recent high throughput genomic studies classified liver cancer into several molecularly distinct subtypes. We thus asked whether AFP activation is related to the molecular classification of liver cancer. Higher serum AFP level was significantly associated with iCluster (Chi-square: 16.86, P = 0.0002) of liver cancer. The iCluster clustering of liver cancer was derived by integrating multiple whole genome platforms, which is supposed to be the most comprehensive and unbiased classification of cancer 16. Specifically, 20 out of 31 liver cancers patients with high serum AFP levels belonged to iClust1, which ranked lowest in prognosis of all three subtypes. These data suggest AFP re-expression is part of systematic changes in liver cancer that define different subtype of tumors.

## Discussion

Aiming at identifying the molecular mechanisms underlying the elevated levels of the serum tumor markers, we performed a pan-cancer analysis of AFP, CEA and PSA to assess the relationship between concentration of serum tumor markers and the expression of their coding genes. Only AFP expression in tumor tissue correlated with its serum levels. In addition, high expression of AFP defined a subtype of liver cancer with distinct gene expression profiles and clinical features. Methylation analysis revealed de-methylation of AFP promoter occurred in some liver cancer tissues, and was significantly related to AFP mRNA expression.

Previous studies suggested that serum AFP levels are mainly controlled at transcriptional level in fetal liver. For example, in normal development of mouse liver, a parallel accumulation of both AFP and albumin mRNAs before birth, followed by a selective nonreciprocal decrease in AFP mRNA after birth, was observed^26^. An interesting finding in our study is that the spectrum of tumor types that produce AFP according to literature matches tumor types with high AFP mRNA expression in TCGA dataset perfectly. This is quite different from other popular serum tumor markers like CEA and PSA, which were expressed at equally high levels in tumor tissue as their normal counter parts. These data confirmed transcriptional control as a key mechanism regulating AFP levels in the serum of the cancer patients.

Liver cancer is a heterogeneous disease. Many molecular subtypes have been proposed for liver cancer 16,27-29, of which the iCluster defined by TCGA is a popular one. The iCluster was derived from a joint analysis of five platforms of genomic data and three major subtypes were derived (iClust1, iClust2, iClust3). We found that AFP was significantly enriched in iClust1 subtype of liver cancer. iClust1 tumors are characterized by younger age, Asian ethnicity, female gender and high tumor grade. In addition, iClust1 patients showed poorest survival among the three subtypes, which is consistent to our analysis showing poor prognosis of AFP high patients^16^ .

Most previous studies investigated AFP alone. What we found in this study suggested aberrant activation of AFP was not an isolated event. It was actually part of the systematic diversion of the transcriptome in the liver cancer cells. More importantly, this diversion in transcription also has clinical significance. The most obvious features of AFP high tumors is its poorer prognosis compared with serum AFP normal tumors. Actually, the inferior prognosis in serum AFP positive tumors has been reported by many other studies already^30^,^31^.

Although the transcriptional control of AFP expression is evident, it's unclear how liver cell cease expressing AFP after birth, and how AFP expression is re-activated in liver cancer. *In vivo* experimental using mouse models revealed that a 7 kb regulatory region upstream of AFP gene was related to the regulation of AFP expression 32. Within this region a tissue-specific promoter, three independent enhancers, and a silencer that is at least partially responsible for AFP gene expression decrease in adult liver have been defined^7^. Of note, the sequence from -200 to the transcription start site of mouse AFP is characterized by tissue-specific promoter activity and contains multiple overlapping binding sites for ubiquitous and tissue-specific transcription factors 33. Our data revealed that de-methylation of a CpG locus in the AFP promoter occurred in some liver cancer tissues, and was significantly related to AFP mRNA expression. Thus, demethylation AFP promoter may be a reason for aberrant AFP expression in some liver cancers.

Evidence suggests that AFP may not only be a biomarker for cancer diagnosis, but also play functional roles in tumorigenesis. AFP can promote cancer cell proliferation through binding AFP receptor (AFPR), activating PI3K/AKT and many other cancer related genes 34-38. Another study found that binding of AFP/AFPR leaded to Ca^2+^ influx, prompting DNA synthesis and enhancing tumor cell proliferation 39. Interestingly, our gene co-expression analysis assigned AFP into M4 gene module, which was significantly enriched with genes related to cell proliferation control.

In conclusion, this study indicates that AFP re-activation is a result of the systematic transcriptome change, which collectively define a molecular subtype of liver cancer. More importantly, high AFP expression is associated with over activation of cell growth or cell cycle control genes. In addition, high AFP expression is related to poor survival in the patients. These data highlights AFP as a biomarker for both liver cancer classification and prognosis.

## Supplementary Material

Supplementary figures and tables.

## Figures and Tables

**Table 1:** Correlation between AFP expression and clinical factors

			AFP low	AFP high		
**Variable**	Total Cases*	**Levels**	**Count1/Mean1**	**Count2/Mean2**	**t/chisq**	**P**
**General information**						
Gender	138	female	34	16	3.28	0.07
		male	73	15		
Age	138		61.5±13.43	58.65±16.03	0.9	0.37
Child pugh	98	A	68	15	1	0.32
		B	10	5		
HBV	138	negative	86	25	0	1
		positive	21	6		
HCV	138	negative	82	28	2	0.16
		positive	25	3		
**Pathology**						
Leukocyte			0.19±0.13	0.2±0.19	-0.34	0.74
Grade	137	G1	16	1	8.07	0.018
		G2	59	13		
		G3	31	17		
Stage	131	I	52	8	5.76	0.056
		II	23	10		
		III/IV	26	12		
Purity	126		0.73±0.21	0.7±0.22	0.67	0.51
**Mutation**						
TP53 mutation	135	no	75	20	0.35	0.56
		yes	29	11		
CTNNB1 mutation	135	no	73	27	2.73	0.099
		yes	31	4		
TERT mutation	136	no	56	21	1.48	0.22
		yes	49	10		
**Molecular classification**						
Paradigm clusters	132	C1	30	3	19.76	0.00019
		C2	16	14		
		C3	21	11		
		C4	34	3		
iCluster	130	1	27	20	16.86	0.00022
		2	37	2		
		3	35	9		

*Note. Only 138 out of 196 patients had information of serum AFP levels and were used in the correlation study. Some patients also lack other clinical information and the total counts of patients may be less than 138.
