# Supplementary material for: Aberrant AFP expression characterizes a subset of hepatocellular carcinoma with distinct gene expression patterns and inferior prognosis
Source: J Cancer. 2020 Jan 1;11(2):403–13. doi: 10.7150/jca.31435 (PMC6930420; doi:10.7150/jca.31435)
Supplement: Supplementary file 1 — Supplementary figures and tables. [file jcav11p0403s1.pdf]

Supplement Figure 1

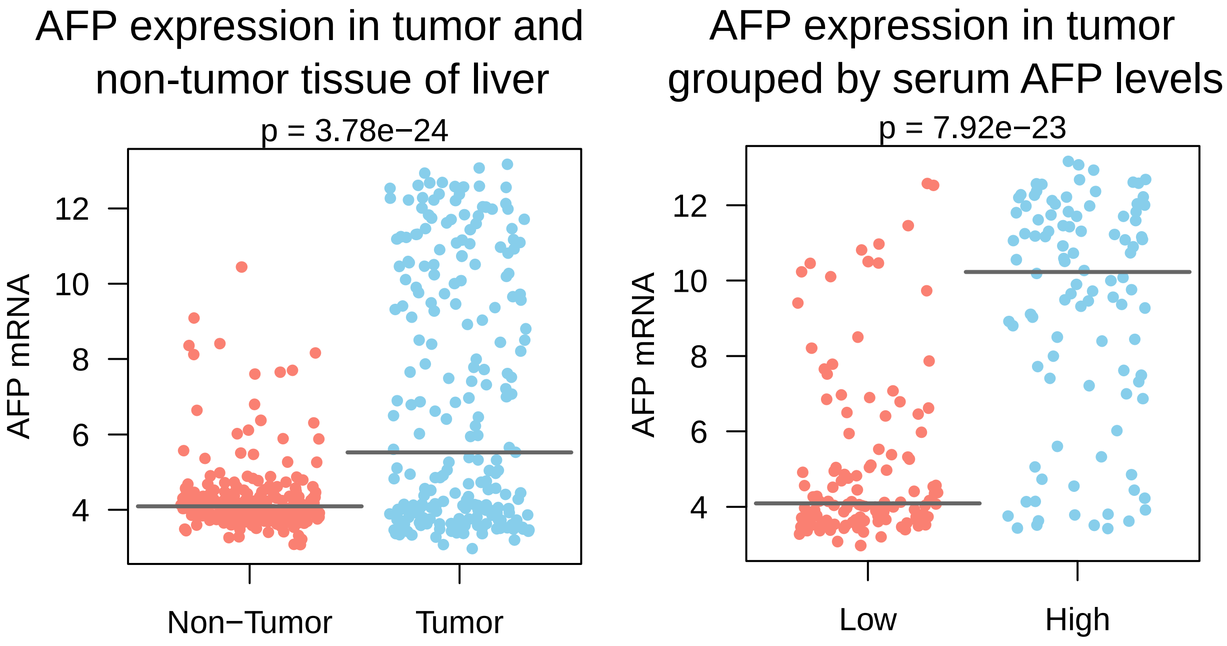

Supplement Figure 1. AFP expression in hepatocellular carcinoma in dataset GSE14520. The plot on the left showed AFP mRNA expression in hepatocellular carcinoma tissue and normal liver tissue. The plot on the right showed AFP mRNA expression in hepatocellular carcinoma tissue grouped by serum AFP levels of the patients. Group means were compared by t-test and the p values were presented above each plot.

Supplement Figure 2

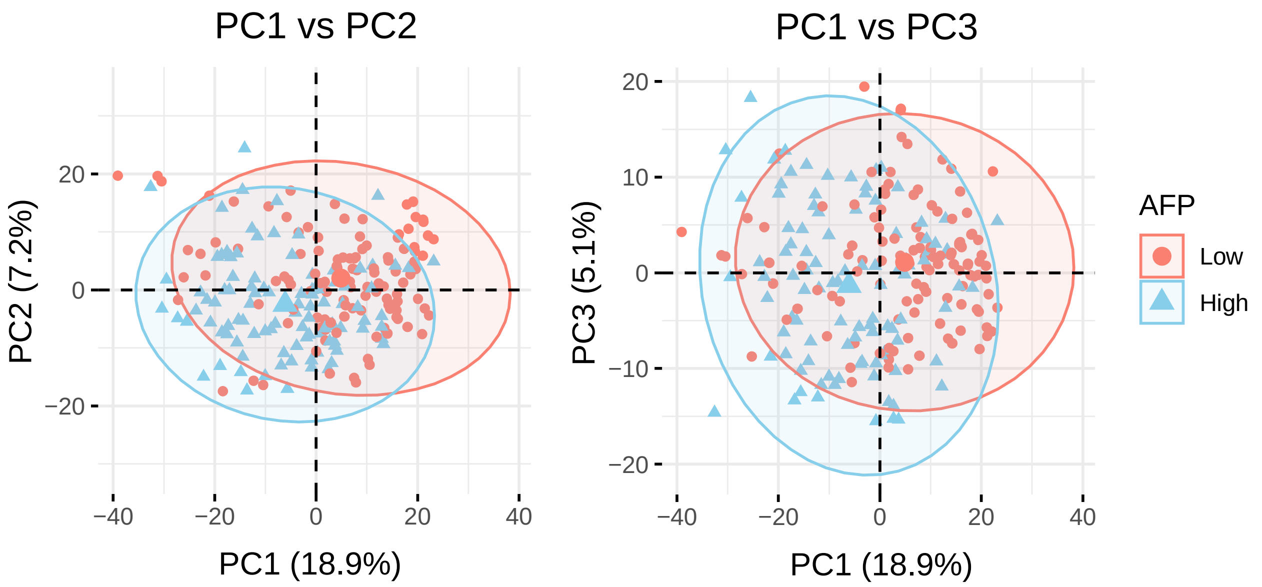

Supplement Figure 2. PCA analysis of liver cancer using whole transcriptome profiling data from dataset GSE14520. Samples were visualized using the first and second principal components (the left plot) or the first and third principal components (the right plot).

Supplement Figure 3

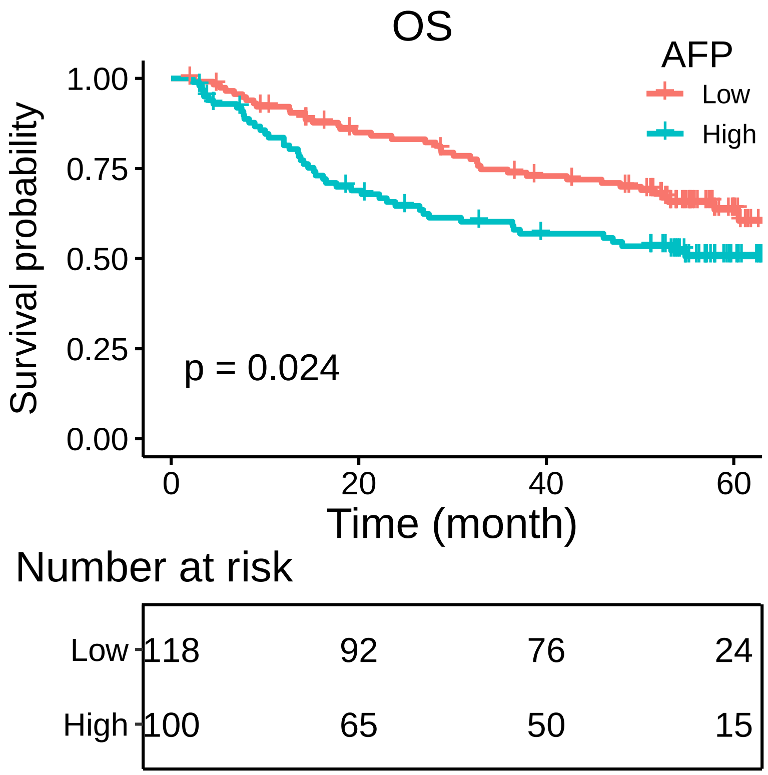

Supplement Figure 3. Prognostic value of AFP in liver cancer in dataset GSE14520. Liver cancer patients were divided into two groups according to their serum AFP levels (use 300 ng/ml as a cutoff value). High serum AFP was associated with poorer overall survival (OS, log rank test: p = 0.024).
